# Cellular Cardiomyoplasty and Cardiac Regeneration

**DOI:** 10.2174/157340308784245748

**Published:** 2008-05

**Authors:** Lakshmana Pendyala, Traci Goodchild, Radhika R. Gadesam, Jack Chen, Keith Robinson, Nicolas Chronos, Dongming Hou

**Affiliations:** Saint Joseph’s Translational Research Institute / Saint Joseph’s Hospital of Atlanta, GA, USA

**Keywords:** Myocardial infarction, heart failure, myoblasts, bone marrow progenitor cells, clinical trials, review.

## Abstract

Despite of vast improvements in treatment, myocardial infarction often leads to heart failure (HF) which remains the leading cause of death in developed countries. Other than heart transplantation, therapeutic options have a limited role in improving out comes in patients with severe HF. It is therefore no surprise that cardiac cell therapy has raised many hopes as a novel therapeutic approach aimed at cardiac myocyte replacement/regeneration termed “cellular cardiomyoplasty”. However, the ideal source, cell type, critical cell number, and mode of application for optimal therapeutic effect have not been defined thus far. Recent observations of the beneficial effect of cell transplantation in animal experiments have generated tremendous excitement and stimulated clinical studies suggesting that this approach is feasible, safe, and potentially effective in humans. Cell-based myocardial regeneration is currently being explored for a wide range of cardiac disease states, including acute and chronic ischemic myocardial damage, cardiomyopathy and as biological heart pacemakers. The main purpose of this article is to review recent literature on the use of various cells for the examination of their *in vitro* cardiogenic potential and their *in vivo* capacity to engraft and improve the functional properties of the infarcted heart.

## INTRODUCTION

Heart failure (HF) is a major and growing public health problem in the United States. Approximately 5 million patients have HF, and over 550,000 patients are diagnosed with HF for the first time each year [1]. The number of HF deaths has increased steadily despite advances in treatment due in part to the better treatment and “salvage” of dysfunctional cardiac tissue in patients with acute myocardial infarction (AMI) [1]. This situation is expected to become worse, with a sharp increase in coronary vascular disease (CVD) in developing countries along with aging world population and it is predicted that 25 million CVD deaths will occur worldwide by 2020 and reach pandemic proportion as projected by the World Health Organization (WHO) [2].

The two primary treatment options available for advanced HF are pharmacologic therapy and cardiac transplantation [3]. Advances in medical therapy have had an important impact on symptom status and short-term survival of patients with moderate to severe HF. The mainstay life-saving drugs are angiotensin-converting enzyme (ACE) inhibitors and beta-blockers. Additional benefits are obtained when angiotensin-receptor blockers or aldosterone antagonists are added [4]. Existing pharmacologic agents have met with only moderate success in patients with class IV HF, and the 1-year survival rate is only 40–50% [5]. Heart transplantation remains the treatment modality with the best outcome with 5-year survival rates of around 65%; however, it is limited by current shortage of donor organs [6]. The success of cardiac transplantation remains further limited by the complications of long-term immunosuppression and the development of allograft CAD. A variety of circulatory support devices have been developed and are being used as bridge to transplant and more recently for destination therapy in patients experiencing end-stage HF who are ineligible for transplantation. The Randomized Evaluation of Mechanical Assistance for Treatment of Congestive Heart failure (REMATCH) trial explored the use of left ventricular assist devices as permanent implants and showed an increase median survival by 7.4 months and improved functional status in comparison with medical management in end-stage HF patients. Though shown to be beneficial, they have high device failure rate and numerous complications, mainly infections and bleeding as a result of necessary anticoagulant therapy [7, 8].

Myocardial infarction (MI), one of the major contributor of HF occurs in approximately 1 million patients annually. Despite optimal medical therapy and aggressive revascularization strategies, MI with left ventricular dysfunction is a lethal condition for 25% of patients over 3 years after the event [9]. Current clinical interventions to minimize these devastating effects range from acute percutaneous coronary intervention (PCI) to administration of drugs such as beta-blockers, which inhibit neurohormonal activation or ACE inhibitors which prevent activation of the renin-angiotensin system. However, these therapeutic measures are not sufficient to prevent left ventricular remodeling and subsequent development of HF.

Cell transplantation is an area of growing interest in clinical cardiology as a potential means of treating patients with MI and/or HF. For HF, the goal of cell therapy is replacement of akinetic scar tissue by viable myocardium in hopes of improving cardiac function along with inhibition of the remodeling process. For MI, the target is to prevent HF by either rescuing the host myocardium or regenerating cardiac cells. Both for the clinician and to the public, the concept of not only preventing the progression and consequence of disease but reversing the disease process by enhancing repair and regeneration of damaged tissues has introduced a new and exciting paradigm to treat cardiovascular disease.

Therefore, the main purpose of this article is to review recent literature on the use of cells for the examination of their *in vitro* cardiogenic potential and their *in vivo* capacity to graft and improve the functional properties of the heart.

## CELLULAR CARDIOMYOPLASTY

Cellular cardiomyoplasty involves myogenic cell grafting within the myocardium to limit any consequences from the loss of contractile function of a damaged left ventricle (LV) [10, 11]. Cell-based cardiac repair offers the promise of regenerating damaged myocardium by rebuilding the injured heart from its component parts. Ideally, transplanted cells would mimic the lost myocytes morphologically and functionally, with the ability to contract and to establish electrical connectivity with the native myocardial cells.

### Definition of Stem Cells

Stem cells are undifferentiated tissue progenitor cells that can proliferate and are defined by their ability to self renew and to form one or more differentiated cell types [12-14]. They can be categorized anatomically, functionally, or by cell surface markers, transcription factors, and the proteins they express. Different populations of stem cells are distinguished by the types of specialized cells that they generate. One clear division of the stem cell family is between those isolated from the embryo, known as embryonic stem cells (ESC), and those in adult somatic tissue known as adult stem cells. Within these categories, stem cells can be further divided according to the number of differentiated cell types they can produce. Totipotent stem cells are able to form all fully differentiated cells of the body and trophoblastic cells of the placenta. The embryo, zygote, and the immediate descendants of the first two cell divisions are the only cells considered to be totipotent [15]. Pluripotent cells can differentiate into almost all cells that arise from the three germ layers, but are unable to give rise to the placenta and supporting structures. At around 5 days after fertilization, ESC that form the inner cell mass of the blastocyst are considered pluripotent. Multipotent stem cells are capable of producing a small range of differentiated cell lineages appropriate to their location and are usually found in adult tissues. Stem cells with the least potential for differentiation are termed unipotent.

## POTENTIAL SOURCES - PRESENT AND FUTURE CELL TYPES

A variety of stem and progenitor cell populations could be used for cardiac repair. Each cell type has its own profile of advantages, limitations, and practicability issues in specific clinical settings. Studies comparing distinct cell types are scarce. The first clinically relevant cells to be proposed as a surrogate for cardiomyocytes were skeletal muscle myoblasts. Bone marrow which is easily accessible is, at present, the most frequent source of cells used for clinical cardiac repair [16] Fig. (**1**). It contains a complex assortment of progenitor cells, including hematopoietic stem cells (HSC), mesenchymal stem cells (MSC) [17], and multipotential adult progenitor cells (MAPC), a subset of MSC [18]. Endothelial progenitor cells (EPC) are isolated from peripheral blood and many times are expanded in culture in ‘endothelium-specific’ medium prior to transplantation into the heart [16, 19]. Other cell population that were investigated include: fat tissue-derived multipotent stem cells [20] multipotential cells from bone marrow or skeletal muscle [21] somatic stem cells from placental cord blood [22], amniotic fluid-derived stem (AFS) cells [23], and cardiac-resident progenitor cells that have a heightened predisposition to adopt the cardiac muscle fate [24, 25]. In each of these newer cases, techniques to isolate and purify these small populations of potent cells will need to be optimized for clinical use. Several researches like Marquette *et al. *also studied the effects of regulatory pathways involved in cardiac development and their utility in reprogramming cardiomyocytes to aid in cardiac protection or repair and found that thymosin b4, a protein involved in cell migration and survival during cardiac morphogenesis, may be re-deployed to minimize cardiomyocyte loss after cardiac infarction [26].

### Embryonic Stem Cells

ESCs are derived from the inner cell mass of blastocyst stage of the embryos; they grow indefinitely in an undifferentiated state while retaining the ability to differentiate to all cell types in the adult body including cardiomyocytes [27]. In culture these cells contract rhythmically [28]. Limited experience with ESC [29-31] indicates that the potential for cardiac repair is greater compared to that of bone marrow cells. Even though ESC show promise as a potential new therapeutic strategy, several questions need to be answered before clinical application of ESC. Assuming they can be produced homogeneously in sufficient numbers, the best method and site to deliver them would still need to be determined. Another important issue is graft rejection. Furthermore, the fate of transplanted ESC or their derivatives would have to be examined in terms of efficacy and safety. Because of unresolved ethical and legal issues, concerns about tumorogenicity and arrhythmogenecity of the cells, and the need to use allogeneic cells for transplantation, ESC have not been investigated broadly and will most likely not be used clinically in the near future.

### Umbilical Cord Stem Cells

Stem cells isolated from umbilical cord blood have been shown to possess a potential for plasticity at least similar to, and perhaps even greater than human adult stem cells; also their differentiation into cardiac myocytes has been demonstrated experimentally [22]. As umbilical cord stem cells can be obtained without the need to sacrifice an embryo, their isolation, use for research purposes and clinical applications are not complicated by the ethical and political issues surrounding the debate over embryonic stem cells.

### Amniotic Stem Cells

DeCoppi *et al*. [23] reported the isolation of a new type of stem cell from amniotic fluid that has many characteristics of ESC without the ethical concerns. AFS cells seem to represent an intermediate stage between embryonic and adult stem cells in terms of their versatility. They are fully undifferentiated and pluripotent.

### Cardiac Stem Cells

Until recently, our perception was that the adult mammalian heart was an organ without regenerative capacity. However, in the past few years, various reports demonstrated the existence of cycling ventricular myocytes both in the normal and pathologic adult heart [32]. Moreover, it has been shown that, in the regions adjacent to the infarcts from patients, 4% of myocyte nuclei expressed the Ki-67 cell proliferation marker. The reentry of myocytes into the cell cycle has been quantified as, respectively, 0.08% or 0.03% for the zones adjacent or distant to the infarcts [33]. In addition, it has also been reported that myocyte hyperplasia contributed to the cardiac hypertrophy perhaps due to the proliferation of cardiac stem cells (CSC) [33, 35]. These newly described stem cells are multipotent, giving rise to endothelial cells, smooth muscle cells, and functional cardiomyocytes. In addition, they supported myocardial regeneration after infarction in a rat model [25]. Future research on CSC will help to answer these questions and may provide the means for efficient heart regeneration. CSCs are implicated in the normal turnover of myocytes, endothelial cells, smooth muscle cells, and fibroblasts. Understanding the mechanisms of cardiac homeostasis would offer the extraordinary opportunity to potentiate this naturally occurring process and promote myocardial regeneration following tissue injury. Smith *et al*. have shown the feasibility of generating human cardiospheres and expanding stem cells from routine endomyocardial biopsy specimens. Human and porcine CSCs can differentiate into electrically functional myocytes *in vitro*. Human CSCs injected into mice lead to myocardial regeneration and functional improvement after infarction [34].

## POSSIBLE MECHANISMS OF ACTION

The mechanisms by which stem cells repair damaged myocardium or lead to improvement in cardiac function are largely unknown, however, the two fundamental activities of stem cells are (a) directly or indirectly improve neovascularization (vasculogenesis, angiogenesis and arteriogenesis) (b) differentiation into cardiomyocytes and formation of myocardial tissue. Functional benefits may also be mediated through paracrine secretion of growth factors or cytokines which indirectly promote survival of cardiomyocytes by inhibition of cardiac apoptosis, and may also lead to mobilization of endogenous progenitor cells, all of which affect remodeling. A small number of stem cells have been demonstrated to fuse with the native dysfunctional myocytes and even differentiate into cardiac myocytes to augment function [36, 37]. So far, a wide range of cell populations have been tested and almost all appear to confer benefit which hints at a possible involvement of various mechanisms. The extent to which these different mechanisms are active may critically depend on the cell type and setting. The ultimate success of cell therapy will rest on its ability to show clinical efficacy rather than on the imputed mechanism [16].

## CELL DELIVERY

The goal of any cell delivery strategy is to transplant sufficient numbers of cells into the myocardial region of interest and to achieve maximum retention of cells within that area. The three most frequently used routes in clinical setting are intracoronary infusion, percutaneous endocardial or direct intramyocardial injection during surgery. Intracoronary infusion requires migration through the vessel wall into the damaged tissue. Some cell type like bone marrow-derived and blood-derived progenitor cells are known to extravasate and migrate to ischemic areas [38], whereas others do not. Satellite cells and mesenchymal cells have been shown to even obstruct the microcirculation in higher doses after intraarterial administration, leading to embolic myocardial damage [39]. By contrast, direct delivery of progenitor cells into scar tissue or areas of hibernating myocardium by catheter or surgical based needle injection may generate relative higher local retention and less systemic distribution. In experimental models, intravenous delivery of EPC has been shown to improve cardiac function after acute myocardial infarction (AMI) [40, 41]. However, homing of cells to noncardiac organs limits the clinical applicability of this approach.

## CLINICAL EXPERINCE

Among the most important issues being addressed at present is identifying the most suitable stem cells for replacing muscle mass and examining which mechanisms might contribute to cell-mediated improvement in cardiac function after MI such that they could be used additionally or alternatively to vital muscle replacement. The experimental evidence that administration of stem cells leads to restoration of myocardial function in models of ischemic cardiac damage is overwhelming and exciting [16, 41]. Because of this success in animal studies, translation into clinical trials started early [42]. The most frequently tested cell types in clinical trials are skeletal myoblasts and bone-marrow or blood-derived progenitor cells. One major pitfall of using autologous cells is that the number of functional stem cells is generally depleted with a markedly reduced proliferation potential in the elderly and in patients with cardiovascular disease.

## SKELETAL MYOBLASTS

Skeletal myoblasts (SM) or satellite cells represent an autologous source of progenitor cells that normally lie in a quiescent state under the basal membrane of mature muscular fibers and normally mediate regeneration of skeletal muscle. Studies indicate that it is feasible to establish and expand myoblast cultures from skeletal muscle biopsies and to obtain target myoblast numbers (10^9^) within 2 to 3 weeks [43]. Myoblasts differentiate into myotubes and multiple lines of evidence now indicate that these cells retain skeletal muscle properties when transplanted into an infarct scar with the exception of rare fusion events between skeletal muscle cells and cardiomyocytes. Although myotubes remain functionally isolated, as they do not couple with resident cardiomyocytes electromechanically and therefore do not beat in synchrony with the rest of the heart, studies in small and large animal models of myocardial infarction have reported beneficial effects of myoblast grafting on both systolic and diastolic performance [44-47]. Part of the protection seems to result from reduced ventricular dilatation, although the complete basis for improved mechanical function is currently unknown. Concern exists about the possible occurrence of serious arrhythmias, a complication which has been shown only in case of SM transplantation [48]. SM might induce arrhythmias by several mechanisms, such as electrotonic stimulation of cardiac cells, electrical heterogeneity of action potentials, increased nerve sprouting, and local tissue injury induced by intramyocardial injection. Since cardiac rhythm disturbances have not been seen with other cell types the latter mechanism is unlikely.

### Clinical Trials

Despite this gap in understanding, myoblasts were the first cell type to be used clinically for cardiac repair owing to their preclinical efficacy, autologous availability, ability to be amplified *in vitro*, and relatively good survival after implantation. To date, SMs have only been used in trials of heart failure, and not for AMI owing to the method of preparation and route of delivery. The use of SMs in humans was first reported by Menasche *et al.* in a single patient with severe ischemic HF [44]. Autologous SMs were implanted into the post infarction scar during coronary artery bypass graft (CABG) to remote myocardial areas. Five months later, there was evidence of contraction and viability in the grafted scar on echocardiography and positron emission tomography along with symptomatic improvement. Other nonrandomized studies have also showed an improvement in symptoms and LV ejection fraction (LVEF) [49-51]. Subsequently, a Phase I non-randomized study of transepicardial myoblast transplantation during CABG showed an improvement in symptoms and LVEF, as measured by echocardiography. Unfortunately, four out of the ten patients in one trial experienced ventricular arrhythmias, necessitating implantable defibrillators [52]. By contrast, no significant ventricular arrhythmias were observed in another Phase I study that recruited 12 patients and again used the transepicardial approach to deliver autologous SM. This study demonstrated a significant increase in LVEF, as well as improved cardiac viability on positron emission tomography (PET) at 3 months, suggesting that the recovery of myocardial function was associated with an increase in functional cell mass [53]. Nevertheless, preoperative use of antiarrhythmic therapy or simultaneous implantation of internal defibrillators has been used to address these possible safety issues. Percutaneous transendocardial route has also been tested and these studies have shown promising improvements in cardiac function while also demonstrating the overall safety and feasibility of this approach [50, 51, 53, 54]. Finally, SMs have been delivered *via* the coronary venous circulation in an attempt to optimize the delivery of these cells to the target tissue and symptomatic improvement was seen without significant arrhythmias [55]. Dib *et al* [52] have reported on 30 patients with a history of ischemic cardiomyopathy, of whom 24 were treated with SM at the time of CABG, whereas the remaining 6 were injected with myoblasts during implantation of a left ventricular assist device as a bridge to heart transplantation. The 24 patients that underwent the cell + CABG procedure were further stratified according to a dose-escalating protocol (12 were divided into four 3-patient groups receiving 1, 3, 10 and 30 x 10^7^, whereas 12 received a fixed dose of 3 x 10^8^). Like in the previous studies, the LVEF increased from 28% to 35% at 1 year (P = 0.02) and to 36% at 2 years (P = 0.01). It was also reported that in some patients, PET and magnetic resonance imaging (MRI) documented improved viability in the cell-injected scar tissue. The results of the Phase II, first randomized, placebo-controlled trial Myoblast Autologous Grafting in Ischemic Cardiomyopathy (MAGIC) included 97 patients at 24 medical centers in Europe undergoing CABG after MI with moderate to severe LV systolic dysfunction. All patients received an implanted cardioverter defibrillator. The study was ended early, since the treatment was not superior to placebo on the primary endpoints of improvement in regional contractility or global function. A significant decrease was documented of LV volumes, a finding which might be clinically relevant since ventricular dimensions are predictors of outcome and long-term follow-up data are awaited [56].

## PROGENITOR CELLS

The bone marrow is known to be an abundant reservoir for many adult stem cells, and bone marrow–derived stem cells (BMC) have been used to treat hematologic disorders for decades. Recent reports have demonstrated that BMC are able to traverse cell lineage boundaries and transdifferentiate into hepatocytes, endothelial cells, skeletal muscle, and neurons upon proper stimulation [57-59]. Although the ability of BMC to transdifferentiate into cardiomyocytes remains highly controversial, much of the recent progress in regenerative cardiovascular research, both in animals and human beings, has been achieved using BMC populations, including hematopoietic stem cells* (*HSC), mesenchymal stem cells (MSC), and endothelial progenitor cells (EPC).

### Hematopoietic Stem Cells

HSC can be isolated from bone marrow cells through selective sorting for a particular set of surface receptors which are lineage negative (Lin−) and positive for stem cell markers (c-kit, Sca-1) [60,61] and represent the prototypical adult stem cell population. The ability of HSC to reconstitute the hematopoietic system of a myeloablated host led to the first clinical application of adult stem cells more than 3 decades ago [62]. Despite the failure of studies to definitely prove differentiation of HSC into cardiomyocytes *in vitro*, several studies in mice have demonstrated the potential of HSC to differentiate into cardiomyocytes or vascular cells after cardiac injury *in vivo* [42, 63, 64].

### Mesenchymal Stem Cells

Within the bone marrow stroma resides a subset of nonhematopoietic cells that have the potential to differentiate into cells of mesenchymal origin [65, 66]. These MSC represent approximately 0.001% to 0.01% of the total nucleated marrow cell population. Immunophenotypically, MSC lack the typical hematopoietic antigens (c-kit, CD45, CD34, CD14) but express specific adhesion molecules (ALCAM/CD44) and antigens (SH2/SH3/SH4/STRO-1) [67, 68]. At first, MSC were thought to contribute solely to the formation of the stromal microenvironment in the bone marrow and maintain HSC survival and function. However, subsequent studies have suggested that MSC are themselves capable of multipotency, with differentiation into chondrocytes, osteoblasts, astrocytes, neurons, skeletal muscle, and notably, cardiomyocytes [69-71].

### Endothelial Progenitor Cells

EPC represent a subset of hematopoietic stem cells that are able to acquire an endothelial phenotype *in vitro* [72-75]. EPC express the hematopoietic stem cell markers CD133 and CD34 and the endothelial marker Flk-1 (VEGFR-2) [74]. EPC can be isolated directly from the bone marrow or from the peripheral circulation.

### Clinical Trials

A number of clinical studies employing progenitor cells have been performed to date with only few randomized, controlled trials [40, 42, 76]. The largest study of cardiac cell therapy reported by Schachinger *et al.* [77], the Reinfusion of Enriched Progenitor Cells and Infarct Remodeling in AMI (REPAIR-AMI) trial, is a multicenter trial of intracoronary infusion of BMC after successful PCI for AMI involving 204 patients. At 4 months, the absolute improvement in LVEF, measured by angiography, was greater among patients treated with BMC than among those given placebo (5.5% *vs*. 3.0%, P = 0.01). Subgroup analysis suggested that the benefit was greatest in patients with the worst LVEF at baseline. This double-blind, randomized and placebo-controlled trial provides the best evidence yet for beneficial effects of BMC after AMI. Enthusiasm is somewhat tempered by the modest size of the effect and by a recent report from the bone marrow transfer to enhance ST-elevation infarct regeneration (BOOST) trial that the relative improvement in LVEF after infusion of BMC at 6 months, as compared with no infusion, was no longer significant at 18 months, suggesting that the main effect was an acceleration of recovery [78]. While data on ventricular function at 1 year are not available for REPAIR-AMI trial, it could be demonstrated that intracoronary administration of BMCs is associated with a significant reduction of the occurrence of major adverse cardiovascular events after AMI including death, myocardial infarction, or necessity for revascularization and rehospitalization for HF compared to patients receiving placebo. 

In contrast, in the smaller Autologous Stem-cell Transplantation in AMI (ASTAMI) trial involving three noninvasive imaging methods, Lunde *et al*. [79] did not find a significant improvement in LVEF at 6 months in the mononuclear BMC group, as compared with the control group. Technical differences in the characteristics or handling of the infused BMC might explain the different outcome. Janssens *et al.* [80] also did not detect an improvement in global ventricular function at 4 months in the BMC group as compared with the control group, although infarct size was reduced and regional wall motion was improved in the BMC group. The identification of features of BMC preparations and of patients that are predictive of a favorable response should help to resolve these discrepancies and to focus future trials. Given the relatively small number of events, this result will require replication in larger cohorts. However, it reinforces the message that BMC infusion is not only feasible but also safe, and it raises the possibility that clinical benefits may exceed the modest improvement seen in ventricular function. These studies provide a realistic perspective on this approach while leaving room for cautious optimism and underscoring the need for further studies.

The Transplantation of Progenitor Cells and Recovery of LV Function in patients with Chronic ischemic Heart Disease (TOPCARE-CHD) trial by Assmus *et al*. [81] evaluated the effects of BMC derived from circulating blood in patients with chronic ventricular dysfunction. In this randomized, crossover trial, the absolute change in LVEF was significantly greater among patients receiving BMC. The groups received the other type of cell in the next phase of the trial, but the result was independent of the order in which the cells were given, suggesting that the BMC effect is somewhat specific. Which quantitative or qualitative differences in the cell populations account for their different effects is currently unknown. Although the benefit observed after BMC infusion was modest (an increase in LVEF by 2.9% points), it is remarkable that any benefit was seen in these patients, who were studied on average more than 6 years after infarction and who were already receiving optimal medical care. The TOPCARE- CHD trial suggests that BMC can have effects beyond simple acceleration of healing after infarction. Whether repeated infusions would yield additive benefits and whether these benefits would persist will be important questions for future trials. Assmus hereby confirms the data by Willerson *et al*., who described for the first time that injection of bone marrow cells, is not only safe but also increases exercise capacity in patients with ischemic cardiomyopathy who were heart transplant candidates [82].

## CARDIAC TISSUE ENGINEERING

A different concept in cardiac regeneration is grafting *ex vivo *engineered heart muscle. This approach may theoretically allow complete replacement of diseased myocardium or reconstitution of cardiac malformations. Large myocardial patches depend critically on metabolic supply, and thus vascularization is crucial. Not only structural but also electrical integration into the host myocardium is necessary. Zimmermann *et al*. [83] have developed a methodology to create engineered heart tissue (EHT) from neonatal rat heart cells, liquid collagen I and Matrigel as well as growth supplements, reconstituted in circular molds and subjected to mechanical strain. Under these conditions, cardiac organoids developed spontaneously and showed contractile as well as electrophysiologic properties of working myocardium. Implantation experiments in healthy rats showed survival, and signs of terminal differentiation of EHT grafts. In a rodent model of myocardial ischemia EHTs integrate and electrically couple to host myocardium display strong vascularization and exert beneficial effects on systolic and diastolic LV function without inducing arrhythmias. This observation is not trivial given the fact that EHTs are not homogeneous heart muscles but organoids consisting of muscle strands, primitive capillaries, fibroblasts, smooth muscle cells and macrophages in a collagen matrix. Although complete reversal of myocardial dysfunction after EHT engraftment was not observed, this study can serve as a proof of principle for a tissue engineering approach in repair of cardiac muscle. However, cardiac tissue engineering is still in its infancy with several important questions that remain in terms of potential clinical applications [83].

Ott *et al.* engineered a biocompatible cardiac extra cellular matrix scaffold with perfusable vascular tree, patent valves and four chamber geometry templates by perfusion decellularization. Reseeding of decellularized heart was done by intramural injection of cardiac derived cells and by perfusion of endothelial cells in vascular conduits. The recellularized construct was functional and drug responsive for 8 days of culture. With sufficient maturation, and given the further ability to reseed its inherent vascular architecture and interior with endothelial cells, this organ could become transplantable either in part or as entire donor heart in end stage HF [84].

## CHALLENGES AND FUTURE

Stem cell treatment of the heart has not been shown to lead to the development of large caliber coronary vessels but rather to capillaries and arterioles by both angiogenesis and vasculogenesis. Therefore, stem cells are either used as adjunct to PCI or CABG or in patients with angiographically proven CAD without viable percutaneous or surgical treatment options. These include patients with diffuse small vessel disease, in-stent restenosis, and chronic total occlusion. It has been estimated that over 100,000 patients may be in this ‘no-option’ group in the US each year [85]. Many alternative approaches have been tested in the past, including transmyocardial laser revascularization, active and passive cardiomyoplasty, gene therapy, surgical ventricular remodeling, coronary endarterectomy and growth factor application, most of which yielded no or very little improvement at best.

The observation that stem cells might augment and assist cardiac regeneration has not only caused debates, but also led to enormous excitement and intense investigations in the rapidly advancing field of cellular cardiomyoplasty. Knowledge created by basic scientists and clinicians, developmental biologists and engineers has led to a better understanding of the molecular signals and cues of cardiac regeneration, cardiopoiesis and cardiomyogenesis and provided us with greater insights into human biology. The use of stem cells and progenitor cells for therapeutic intervention in cardiovascular disease holds not only great promise, but also harbors significant controversy. 

Despite the advances that have been made in this broad area, it is important to emphasize that there are still fundamental questions that need to be addressed both experimentally and clinically regarding potential features of cell repair. The most eminent unresolved issues are; cell delivery, optimization of cell retention, distribution, the best route of delivery, time of transplantation, cell type, cell number, and viability of grafted cells. Strategies to genetically modify stem cells aimed to improve survival have been employed [86]. Hill *et al.* [87] observed a strong correlation between the number of circulating EPCs and the subjects’ combined Framingham cardiovascular risk factor score. Therefore, with the onset of disease (or the presence of risk factors), the relevant cells appear to decrease in number and lose their reparative function. Despite the high number of stem cell studies performed, there is still no consensus on the optimal/minimal cell number required to achieve any effect. In fact in a few clinical studies, investigators used a cell number in clinical trial that would barely suffice to treat a mouse heart [82]. While functional improvement of the infarcted heart by stem cells has been recognized even by fierce disbelievers in cellular cardiomyoplasty, the way by which stem cells regenerate the heart are not yet elucidated. A surprisingly wide range of nonmyogenic cell types improves ventricular function, suggesting that benefit may result in part from mechanisms that are distinct from true myocardial regeneration [88, 89]. Future trials should be randomized, controlled and designed and powered to examine clinical end points and patients should be followed over the long term and for both beneficial and adverse effects.

Considerable work needs to be done before cell based therapy can be used routinely in the clinical setting for people. We are confident, however, that the exciting approach of cellular cardiomypoplasty will lead to an effective clinical therapy and thus has the potential to improve the health of millions of people worldwide each year.

## Figures and Tables

**Fig. (1):**
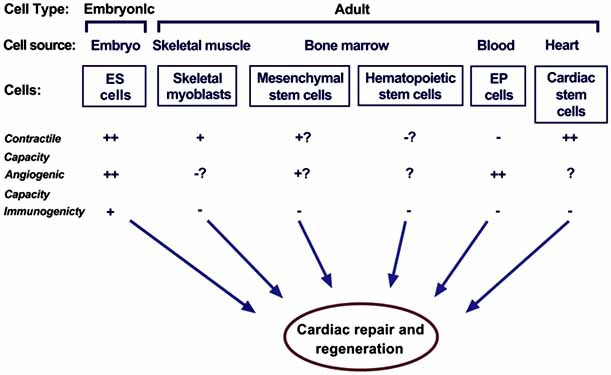
Potential stem cells for cellular cardiomyoplasty

**Table 1:** Selected Clinical Trials of Autologous Skeletal Myoblasts in Cardiomyopathy

Study	Design(n)	Cell type	Route	Follow-up	Outcomes
Menasche *et al.* (2003)	Series (10)	Myoblasts	Epicardial During CABG^5^	5-17.5 months	Improved NYHA^3^ Improved LVEF^1^
Herreros *et al.*(2003)	Series (12)	Myoblasts	Epicadial During CABG^5^	3 months	Improved LVEF^1^
Simniak *et al*. (2004)	Series (10)	Myoblasts	Epicadial During CABG^5^	6 months	Improved LVEF^1^
Dib *et al.* (2005)	Series (30)	Myoblasts	Epicadial CABG^5^+LVAD^2^	2 years	Improved LVEF^1^
MAGIC (2006)	RCT^4^, double blind	Myoblasts	Epicadial During CABG^5^	Early termination	No effect on EF + LV remodeling

1 LVEF, Left ventricular ejection fraction;

2 LVAD, left ventricular assist device;

3 NYHA, New York Heart Association functional class;

4 RCT, randomized clinical trial;

5 CABG, coronary artery bypass grafting

**Table 2:** Selected Clinical Trials of Bone Marrow-Derived Stem Cells

Study	Design(n)	Cell type	Route	Outcomes
BOOST	RCT, open label (60)	BM-MNC^1^	Intracoronary	Improvement of LVEF^5^ at 6 months
TOPCARE-AMI	Randomized (59)	BM-MNC^1^*vs*. CPC^2^	Intracoronary	Improved LVEF^5^ and perfusion
Janssens *et al.*	RCT, double blind (67)	BM-MNC^1^	Intracoronary	Reduced infract size
ASTAMI	RCT, double blind (100)	BM-MNC^1^	Intracoronary	No benefit
REPAIR-AMI	RCT, double blind (204)	BM-MNC^1^	Intracoronary	Improved LVEF^5^
REVIVAL-2	RCT, double blind (114)	G-CSF^6^ and PBSC^3^	Mobilization	No effect on LVEF^5^
TOPCARE-CHD	RCT, cross over (75)	BM-MNC^1^*vs*. CPC^2^	Intracoronary	Improvement of LVEF^5^
MAGIC Cell-3-DES^4^	RCT (96)	G-CSF^6^*vs*. G-CSF^6^ + PBSC^3^	Intracoronary	Improvement of LVEF^5^

1 BM-MNC, Bone marrow mononuclear cell;

2 CPC, circulating progenitor cell;

3 PBSC, peripheral bone marrow stem cells;

4 DES, Drug-eluting stent;

5 LVEF, Left ventricular ejection fraction;

6 G-CSF, Granulocyte Colony stimulating factor
